# Clinical Characteristics To Guide The Extent Of Ablation In Paroxysmal AF Patients: Discovering An Old Science

**Published:** 2009-05-15

**Authors:** Anthony G Brooks, Narayanan Namboodiri, Prashanthan Sanders

**Affiliations:** Cardiovascular Research Centre, Department of Cardiology, Royal Adelaide Hospital and the Disciplines of Medicine and Physiology, University of Adelaide, Adelaide, Australia

**Keywords:** atrial fibrillation, atrial fibrillation burden, pulmonary vein isolation, catheter ablation

The last decade has seen an unprecedented expansion in the tools, techniques and patients undergoing primary ablation of atrial fibrillation (AF). Initial series reported selected patients with high ectopy burden that allowed targeting of the arrhythmogenic sites [1-3]. However, with the expansion of the patient population undergoing treatment and the available strategies, our challenge remains identifying how much is enough for a given patient. While limited ablation may result in limited success, extensive ablation improves success rates, but potentially at the cost of greater complications.

Pulmonary vein (PV) isolation is now established as the corner stone of ablation strategies for AF [4]. It is an effective treatment for the majority of paroxysmal AF patients. However, in 30-40% of paroxysmal AF and almost all patients with persistent or permanent AF, additional substrate modification is required to improve outcomes. Several substrate modification strategies have been proposed. Linear and electrogram based ablation (complex fractionated or high frequency) are the most common approaches to substrate ablation. 

Empirical linear ablation of the left atrium (LA) roof or mitral isthmus in all paroxysmal AF patients has demonstrated significant improvements in clinical outcomes [5,6]. However, substrate ablation in whatever form, is associated with additional tissue damage, increased power delivery, increased fluoroscopy and procedure time, greater risk of collateral injury and alterations in normal activation that may potentially be pro-arrhythmic [6,7]. It is clear that the empirical application of substrate modification would expose a significant proportion of patients to additional ablation and resultant collateral injury without clinical need.  A predictive tool to select patients that would benefit from additional substrate ablation and to accurately isolate those that only require PV isolation is required to maximise efficacy whilst minimising risk of additional ablation in paroxysmal AF.

AF inducibility has been used to predict the presence of abnormal substrate to guide the need for further ablation. Inducibility after PV isolation has been associated with poor outcome in paroxysmal AF patients and has been used to direct additional ablation in this cohort [8,9]. Non-inducible patients have 5 times the probability of success compared to those who remained inducible  [8]. Jais et al prospectively evaluated a tailored ablation strategy guided by AF inducibility after each stage of ablation. In this study, PV isolation rendered 57% of paroxysmal AF patients non-inducible. In the remaining, additional ablation was undertaken sequentially with one or two linear lesions within the left atrium rendering 93% of patients non-inducible. At follow up of 18 months, 91% of patients were arrhythmia free without antiarrhythmic drugs. This success rate was greater than that observed with empirical linear ablation in all paroxysmal patients [5,6], whilst saving 57% of individuals from being exposed to substrate modification. However, it was still unclear how many of the inducible patients would have achieved success without additional ablation. Oral and colleagues [10] assessed the efficacy of additional ablation in inducible patients by randomising this subset of patients to 'no additional ablation' or 'further substrate modification'. Substrate modification improved the clinical outcome from 67% to 86%. Taken together, these studies suggest that inducibility after PV isolation is highly sensitive for detection of patients that require additional ablation, but is a poor tool to isolate patients who will succeed with PV isolation alone. In other words, inducibility testing after PV isolation marginally improves over empirical substrate ablation to all paroxysmal AF patients. On the flip side, *non*-inducibility after PV isolation is reasonable for detecting patients who will succeed with PV isolation alone [8]. In an attempt to provide a pre-procedure estimate of PV isolation success, Rotter et al.  [11] performed a multivariate analysis to determine the clinical features that were predictive of non-inducibility after PV isolation and reported that the longest AF episode of < 48 hours, an LA largest diameter of < 57 mm and an absence of structural heart disease were all independently predictive. Hence, these baseline characteristics may be considered to be associated with limited substrate involvement in paroxysmal AF.

When a continuous variable is categorised, data analysis is simplified, but potentially important information is lost. Paroxysmal, persistent and permanent AF categories allow for clear demarcation of important clinical and sub-clinical characteristics associated with AF; however, in doing so, it may over simplify the continuum of arrhythmia severity that is modulated by the presence or absence of various co-morbid diseases and sub-clinical factors. Ablation approach based purely on these clinical categories is likely to underestimate the requirement of ablation to achieve clinical success in some patients and overestimate ablation requirements in others. A more sensitive measure of arrhythmia burden or amount of arrhythmia sustaining substrate is therefore required to guide ablation to *maximise benefit for minimal ablation*. The type of AF has been previously reported to predict ablation outcomes in the general AF population   [12,13] and longest duration of AF in paroxysmal patients has predicted non-inducibility after PV isolation [11]. Total time spent in symptomatic AF 3 months prior to the procedure is a new variable proposed in this issue of the journal to assess the level of substrate involvement in paroxysmal AF.

Berkowitsch and colleagues [14] describe a simple and seemingly effective methodology to separate paroxysmal AF patients who have a poorer outcome with PV isolation and as such, this group may be targeted for additional substrate modification to effectively treat their arrhythmia in future. Using longitudinal and short axis LA diameters and self-reported arrhythmia burden diaries for 3 months prior to the procedure the authors developed an equation to predict paroxysmal AF patients who would perform poorly after PV isolation using either of three vein isolation techniques. Importantly, the different isolation methodologies did not affect the final outcome in the sample patients and were therefore grouped for the predictive analysis. Given the bias introduced by reporting sensitivity and specificity values derived from the same cohort on which they were developed, Berkowitsch et al. developed the prediction equation in one half of their cohort (n=122) and validated it on the other half (n=122). Using ROC curve analysis on the test group a long axis LA diameter >  60 mm (HR=1.5) and a symptomatic AF burden >  500 hours in 3 months (HR=2.0) were independently predictive of a poorer outcome. After dichotomising patients at 500 hrs (~20% AF burden in 3 months) the equation was able to correctly predict PV isolation failures 69% percent of the time and successes on 61% of occasions. These data demonstrate that the proposed cut-off has potential to select patients to undergo additional ablation (with 42% sensitivity), with only a minimal safety cost by adding ablation to 7% of patients who would have had success with PV isolation alone. Assuming that additional substrate ablation has absolute efficacy in the selected patients, this variable has the potential to increase clinical success from the observed 59%, to a postulated 76%.  Furthermore, the cut-point could be modified to increase the sensitivity of test to correctly predict patients who require additional ablation (by lowering the disease burden threshold) at the sacrifice of applying additional ablation to a greater proportion of successful PV isolation patients. Symptomatic AF burden seems to be less sensitive, but much more specific than post PV isolation inducibility testing and may therefore be an important characteristic to tailor ablation in paroxysmal AF and increase the clinical success of ablation beyond 80%, whilst minimising ablation in the majority of paroxysmal AF patients.

## Conclusion

The study of Berkowitsch et al.  [14] demonstrates that not all paroxysmal AF patients respond to PV isolation alone and that some, with greater AF burden or enlarged long axis left atrial diameters are at significantly greater risk of AF recurrence in the two years following their procedure. Applying these parameters to determine the requirement of substrate ablation has the potential to increase the efficacy of ablation for paroxysmal AF patients, without unnecessarily ablating patients that do not require substrate modification. The proposed cut-off AF burden (500 hr/3 month) would accurately select 42% of the patients who do not succeed with PV isolation alone (and hence could be targeted for substrate ablation), whilst only misclassifying 7% of patients to receive substrate ablation unnecessarily. 

In future, multivariate equations may be able to predict the ideal ablation approach to provide a clinical effect using more sensitive clinical characteristics and hence, *minimize ablation for maximal outcome*.
